# Cognitive Decline in Parkinson’s Disease: A Subgroup of Extreme Decliners Revealed by a Data-Driven Analysis of Longitudinal Progression

**DOI:** 10.3389/fpsyg.2021.729755

**Published:** 2021-09-09

**Authors:** Sara Andersson, Maria Josefsson, Lars J. Stiernman, Anna Rieckmann

**Affiliations:** ^1^Neuro-Huvud Halscentrum, Region Västerbotten Hospital, Umeå, Sweden; ^2^Umeå Center for Functional Brain Imaging, Umeå, Sweden; ^3^Department of Integrative Medical Biology, Umeå University, Umeå, Sweden; ^4^Department of Statistics, Umeå School of Business, Economics and Statistics, Umeå, Sweden; ^5^Center for Demographic and Ageing Research, Umeå, Sweden; ^6^Department of Radiation Sciences, Umeå University, Umeå, Sweden; ^7^The Munich Center for the Economics of Aging, Max Planck Institute for Social Law and Social Policy, Munich, Germany

**Keywords:** cognitive decline, cluster analysis, longitudinal, Parkinson’s disease, subtypes

## Abstract

Cognitive impairment is an important symptom of Parkinson’s disease (PD) and predicting future cognitive decline is crucial for clinical practice. Here, we aim to identify latent sub-groups of longitudinal trajectories of cognitive change in PD patients, and explore predictors of differences in cognitive change. Longitudinal cognitive performance data from 349 newly diagnosed PD patients and 145 healthy controls from the Parkinson Progression Marker Initiative were modeled using a multivariate latent class linear mixed model. Resultant latent classes were compared on a number of baseline demographics and clinical variables, as well as cerebrospinal fluid (CSF) biomarkers and striatal dopamine transporter (DAT) density markers of neuropathology. Trajectories of cognitive change in PD were best described by two latent classes. A large subgroup (90%), which showed a subtle impairment in cognitive performance compared to controls but remained stable over the course of the study, and a small subgroup (10%) which rapidly declined in all cognitive performance measures. Rapid decliners did not differ significantly from the larger group in terms of disease duration, severity, or motor symptoms at baseline. However, rapid decliners had lower CSF amyloidß42 levels, a higher prevalence of sleep disorder and pronounced loss of caudate DAT density at baseline. These data suggest the existence of a distinct minority sub-type of PD in which rapid cognitive change in PD can occur uncoupled from motor symptoms or disease severity, likely reflecting early pathological change that extends from motor areas of the striatum into associative compartments and cortex.

## Introduction

Parkinson’s disease (PD) is a neurodegenerative disorder characterized by loss of midbrain dopamine neurons and accumulation of Lewy Bodies. Motor disturbances are the dominant symptoms of PD. However, non-motor symptoms such as cognitive impairment are common and strongly affect quality of life in patients and caregivers.

Deficits in global cognitive functions are often subtle in the early stages of the disease but a diagnosis of dementia is common as the disease progresses to an advanced stage (Barone et al., 2011; Aarsland et al., 2017; Schapira et al., 2017). The presence of cognitive impairment or other non-motor symptoms is a key criterion for the definition of separable subtypes of PD (Marras and Chaudhuri, 2016). State-of-the-art classifications separate a mild motor-predominant subtype, capturing the majority of patients, from diffuse-malignant and intermediate subtypes who exhibit non-motor symptoms including cognitive impairment (Fereshtehnejad et al., 2015, 2017). Such classifications aid in better understanding heterogeneity in PD pathophysiology and to tailor individual treatment optimally.

Regardless of using data-driven (Lewis et al., 2005; van Rooden et al., 2011; Dujardin et al., 2013; Fereshtehnejad et al., 2017; Campbell et al., 2020) or hypothesis-driven (Muslimovic et al., 2005; Elgh et al., 2009; Reid et al., 2011) methods for subtyping PD, a common approach has been to use data from a single assessment, often early in the disease, as the basis for grouping. This approach is sensible if identifying heterogeneity at the time of diagnosis can provide information on subsequent disease progression and survival for a clinician. However, from these analyses, it remains unclear whether distinct “between-person” cognitive profiles in PD at baseline are the best predictors of cognitive decline across time. For example, two patients that do not differ in their cognitive performance or motor symptoms in the first year of the disease may decline at different rates over subsequent years. A useful alternative approach may therefore be to identify heterogeneity in cognitive change across time, and then, in a second step, try to identify predictors of diverging trajectories at baseline.

In the current study, we explore whether longitudinal trajectories of cognitive decline across multiple domains in PD can be described in terms of meaningful latent subgroups. Posing few *a priori* assumptions, we utilize a multivariate latent class linear mixed model (MLCLMM) approach (Lai et al., 2016; Proust-Lima et al., 2017). The model considers multiple variables at once (here: different cognitive domains) and identifies latent subgroups in the data in terms of longitudinal change. To foreshadow the main results, this analysis revealed the existence of a small, homogeneous group of *de novo* PD patients (10%), who showed rapid decline of global cognitive functions (executive domain, memory, and visuospatial ability) over 5years in the early stage of disease. In order to characterize early risk factors of rapid cognitive deterioration in PD, we compared the rapid decliners to the rest of the sample on a number of baseline demographics and a wide range of behavioral clinical assessments. Baseline biomarkers were selected on the basis of *a priori* relevance for cognitive impairment in PD and related neurodegenerative disorders and included dopamine transporter (DAT) loss in caudate and putamen (e.g., Pasquini et al., 2019), APOE4 genotype (e.g., Tan et al., 2021), and levels of α-synuclein, amyloidß42, and tau from cerebrospinal fluid (CSF; e.g., Siderowf et al., 2010; Kang et al., 2016).

## Materials and Methods

### Participants

Data used in the preparation of this article were obtained from the Parkinson’s Progression Markers Initiative (PPMI) database (Marek et al., 2018; www.ppmi-info.org/data). For up-to-date information on the study, visit www.ppmi-info.org. PPMI was conducted in accordance with the Declaration of Helsinki and the Good Clinical Practice (GCP) guidelines after approval of the local ethics committees of the participating sites. Informed consent was obtained from each subject.

At enrolment, PD patients had received a diagnosis no longer than 2years before and were not taking PD medication. DAT status was performed with Iodine-123-labeled ioflupane single-photon emission computerized tomography imaging for the DAT (Darcourt et al., 2010; DaTSCAN) at the first screening visit. Patients below age 50 were also excluded to avoid cases of early onset PD.

An age-, sex-, and education- matched healthy control (HC) group was free of neurological disease, no first-degree family member with PD and used no medication that might interfere with DAT imaging. For both patients and healthy controls, we included only participants with at least two scheduled visits. Data from unscheduled follow-ups outside the original yearly follow-ups were not included in this study. Following these criteria, the final sample size included in the present study were 349 PD patients with an average disease duration of 6.72months (*SD*=6.63) at baseline and 145 HCs. The data were downloaded from the PPMI database on 28 September 2020. At this point, individuals had undergone up to 5 annual follow-up visits.

### Cognitive Assessment

At baseline and at each annual follow-up visit, a comprehensive neuropsychological battery was administered to all subjects by trained personnel as part of the PPMI testing procedure.

For the current study, the core five cognitive tests of the neuropsychological assessment in PPM were considered: (1) the WMS-III Letter-Number-Sequencing Test (Wechsler, 1997), (2) a Symbol Digit Modalities Test (Smith, 1973), (3) the Benton Judgement of Line Orientation Test (Benton et al., 1978), (4) a semantic fluency test (Gladsjo et al., 1999; animal naming), and (5) the Hopkins Verbal Learning Test-Revised (Benedict et al., 2010; alternate forms were used at follow-ups). We obtained the age-corrected, and where available, education-corrected, and normed cognitive scores (T and scale points) from the database and then transformed them into *z*-scores for each test. Further, where appropriate, cognitive domain composite scores were created based on the average of *z*-scores: An executive function (EF) composite included the average of *z*-scores from symbol digit modalities test, letter-number sequencing and semantic fluency, and memory composite included z-scores from immediate recall and delayed recall in the learning test. For the visuospatial domain, only the line orientation test was available. Patients were asked not to take their PD medication on the day of each annual study visit.

### Demographics and Clinical Variables

To identify early markers of different cognitive trajectories, a number of demographic, clinical variables and biomarkers (section “Genetic and CSF Biomarkers”) were used to compare patient sub-groups at baseline (section “*Post-hoc* Explorations”). Demographics included age, sex, and education in years. To evaluate motor impairment in the patients, The Movement Disorders Society Unified Parkinson’s Disease Rating Scale (MDS-UPDRS) part III was used (Goetz et al., 2008). A mean tremor score and a mean postural instability and gait difficulties (PIGD) score were calculated from the respective items. Scores for a tremor dominant subtype (TD) and an akinetic-rigid subtype were then calculated according to the criteria: If mean tremor score/PIGD score≥1.15, OR if PIGD score=0 and Tremor score>0, then subject is TD. If ratio≤0.9, then subject is PIGD (Stebbins et al., 2013). As an indicator of disease stage/severity, the Hoehn and Yahr stage was included (Hoehn and Yahr, 1998). Part II total score of the MDS-UPDRS was used as an additional indicator of motor problems in daily living. Disease duration was calculated as the number of months between diagnosis and enrolment into the study.

Movement Disorders Society Unified Parkinson’s Disease Rating Scale score from part I was used as a general indicator of non-motor aspects of daily living associated with PD. In addition, cognitive impairment was assessed with the brief cognitive screening instrument montreal cognitive assessment (MOCA) (Nasreddine et al., 2005, adjusted for education); sleep disorder was assessed by the rapid eye movement (REM) Sleep Behavior Disorder Questionnaire (Stiasny-Kolster et al., 2007). Olfactory dysfunction with the University of Pennsylvania Smell Identification Test identified hyposmia as a score below the 15th percentile for the patient’s age and sex group (Jennings et al., 2014). Psychiatric features associated with PD were assessed with the short version of the Questionnaire for Impulse-Compulsive Disorder (QUIP; Weintraub et al., 2013), the 15-item Geriatric Depression Scale (GDS-15; Sheikh and Yesavage, 1986) and the 40-item psychiatric State-Trait Anxiety Inventory (STAI). The QUIP was used to indicate the total number of compulsive behaviors (max 7). The 40 items from the STAI were summed into a mean state and a mean trait anxiety score with a range of 20–80 and a higher score indicating greater anxiety (Spielberger et al., 1970).

For the current study, established cut-offs were used to separate normal from abnormal non-motor functions where available. A cut-off of 26 was used for the MOCA to indicate cognitive impairment vs. no cognitive impairment according to Nasreddine et al. (2005). The REM Sleep Behavior Disorder Questionnaire Score was dichotomized into REM sleep disorder (yes-no) using a cut-off of 5 (e.g., Nomura et al., 2011). For the GDS, prior research in PD patients has indicated that a cut-off score of <5 accurately distinguishes depressed from nondepressed patients in PD (Weintraub et al., 2006). Missing data for all questionnaires and clinical assessments were <1%.

### Genetic and CSF Biomarkers

APOE4 genotype and levels of CSF biomarkers were obtained as processed values from the PPMI database. CSF data were available for baseline and follow-up timepoints 1, 2, and 3 of the current study. Briefly, APOE genotyping followed the methods by Livak (1999). Taqman Assays were used per manufacturers protocol to genotype two non-synonymous single nucleotide polymorphisms (SNPs), rs429358 (APOE-C112R) and rs7412 (APOE-R158C), in each patient sample in order to distinguish between APOE ε2, ε3, and ε4 alleles. Patients were grouped into those having at least one ε4 allele vs. the rest. Genotype was missing for 37 patients (10.6%).

The measurements of α-synuclein, amyloidß42, total (t)-tau, and phosphorylated tau 181 (p-tau) levels in CSF are described in detail in Kang et al. (2016). CSF was collected by standard lumbar puncture. α-synuclein, amyloidß42, t-tau, and p-tau were measured by INNO-BIA AlzBio3 immunoassay (Innogenetics Inc.). α-synuclein was measured by enzyme-linked immunosorbent assay. Missing CSF data was 1.7% at baseline, 15.92% at follow-up 1, 17.57% at follow-up 2, and 34.84% at follow-up 3.

### Dopamine Transporter Imaging

In order to investigate the association between cognitive decline and regional neuropathology, i.e., dopaminergic dysfunction, longitudinal DaTSCAN-data were included in the current study. DAT-data were quantified as Striatal Binding Ratios (SBR), a measure of DAT density computed as (target region/reference region)-1, with the target region being the DAT-rich striatum (consisting of caudate and putamen), and a reference region with negligible specific DAT binding, the occipital lobe. For the purposes of this study, the SBR values for right and left caudate and right and left putamen were downloaded from the database and averaged across hemispheres. In order to match the DaTSCAN data as closely as possible with the neuropsychological testing, DaTSCAN data were obtained from the screening visit (mean difference in months to baseline cognitive assessment=0.82, *SD*=1.06; available for 338 patients), and from the scans that were conducted in conjunction with annual follow-up visits 1 (mean diff=0.61months, *SD*=0.49; *N*=298), 2 (mean diff=0.62months, *SD*=0.58; *N*=294), and 4 (mean diff=0.58months, *SD*=0.62; *N*=249).

### Statistical Analysis

#### Identification of Latent Sub-Groups

Statistical analyses were performed in R version 3.6.2 and R studio version 1.2.5033. To identify distinct patterns of longitudinal change across multiple cognitive domains, a MLCLMM was used, implemented in the *multlcmm* function in the R package *lcmm* (Proust-Lima et al., 2017). The MLCLMM is an extension of the linear mixed effects model by latent class analysis for multiple outcome variables simultaneously. The algorithm partitions the population into heterogeneous subgroups (latent classes) based on their longitudinal trajectories across multiple outcomes, i.e., their longitudinal performance across multiple cognitive domains. *Multlcmm* provides a maximum likelihood estimation of MLCLMM using an iterative procedure. The three composite domain scores described in the previous section (Executive Composite, Memory Composite, and Visuospatial Score) were included as multivariate outcome variables in the model. The fixed effects were intercept and timepoint, where the timepoint was added as a factor (baseline, first follow-up, second follow-up etc.) in order to allow for non-linear trajectories such as accelerated decline at later test occasions or initial practice effects. Random effects for subject and timepoint were modeled to account for within-subject correlations between repeated measurements (in other words, controlling for baseline when estimating change).

The number of latent subgroups is unknown and needs to be pre-specified before each model estimation procedure. In this study, we used the Bayesian Information Criterion (BIC) to select the best-fitting number of latent subgroups between 1 and 4. BIC is the most commonly used method and has shown good performance when selecting the best fitting latent class model (Nylund et al., 2007). For the best-fitting solution, entropy was computed as an index of the strength of class separation according to Ramaswamy et al. (1993), where an entropy value close to 1 indicates a good separation of classes.

#### *Post-hoc* Explorations

Visual inspection of mean performance at each timepoint for each class suggested linear effects in rates of change. *Lme4* (Bates et al., 2015) was used to fit separate linear mixed effects models for confirmation and illustration of each cognitive domain, with class, time (timepoint as numeric), and their interaction as fixed effects and subject and time as random effects. For comparison, motor function based on the UPDRS score part III was analyzed in the same way.

In order to identify predictors of cognitive decline at baseline, *post hoc* explorations of differences in demographics, clinical variables, APOE4 group, CSF markers, motor symptoms, and disease severity were performed using Student’s *t*-tests, and where appropriate chi-square tests. In addition, comparisons were made between the whole PD group and HC group at baseline for descriptive purposes where applicable. *Post-hoc* group comparisons were interpreted at a Bonferroni-adjusted value of *p*<0.05, adjusted for 22 different tests (*cf*. Table 1). Finally, CSF biomarker levels and DAT data for caudate and putamen were modeled as linear mixed effects models in the patients, with class, time, and their interaction as fixed effects and subject and time as random effects.

**Table 1:** *Post-hoc* linear mixed effects models.

Domain	Effect	Estimate	*SE*	*p*
Comparison of PD class 1 vs. PD group 2				
EF	Class	−0.10	0.10	<0.001
	Class*Time	−0.23	0.02	<0.001
Memory	Class	−0.86	0.17	<0.001
	Class*Time	−0.25	0.04	<0.001
Visuospatial	Class	−0.77	0.16	<0.001
	Class*Time	−0.10	0.04	0.005
Motor function	Class	2.82	1.63	0.08
	Class*Time	1.34	0.50	0.008
Comparison of PD class 1 vs. HC				
EF	Class	0.17	0.01	0.002
	Class*Time	0.02	0.00	0.135
Memory	Class	0.32	0.01	<0.001
	Class*Time	0.00	0.00	0.966
Visuospatial	Class	−0.02	0.09	0.831
	Class*Time	−0.01	0.02	0.603
Motor function	Class	−17.92	0.08	<0.001
	Class*Time	−2.18	0.20	<0.001

EF, Executive Functions.

In order to test for change-change associations between cognitive decline and loss of DAT binding, individual slopes were fitted to the cognitive performance and DAT data for each patient and then correlated.

## Results

At baseline assessments, patients were on average 63.64years of age (*SD*=7.50), with 15.54 (*SD*=2.99) years of education. Consistent with higher prevalence of PD in men, 65% were male. Out of 349 PD patients, 262 (75%) were retained until the last timepoint. Missing values were below 3% across all of the obtained cognitive measures. For comparison, matched healthy controls were 63.96 (*SD*=7.74) years of age at baseline, with 15.91 (*SD*=2.83) years of education, and 65% men.

### Latent Class Analysis Identifies a Sub-Group of Rapid Cognitive Decliners in PD

Fitting of a MLCLMM to the cognitive data suggested that longitudinal cognitive decline in the patient sample was best described by two latent groups (as indicated by the lowest BIC statistic (Table 2)). The two-class solution split the sample into one larger subgroup with 90% of the PD participants (Class 1. *N*=315) and one smaller subgroup, with 10% of the participants (Class 2. *N*=34, entropy=0.90). The worse-fitting three-and four-class solutions further divided the larger group but retained the smaller subgroup.

**Table 2:** Number of follow-up visits (max 5) by class, in percent.

	1	2	3	4	5
Class 1	4.44	3.49	6.03	9.52	76.51
Class 2	0	5.88	8.82	23.53	61.77

**Figure 1:**
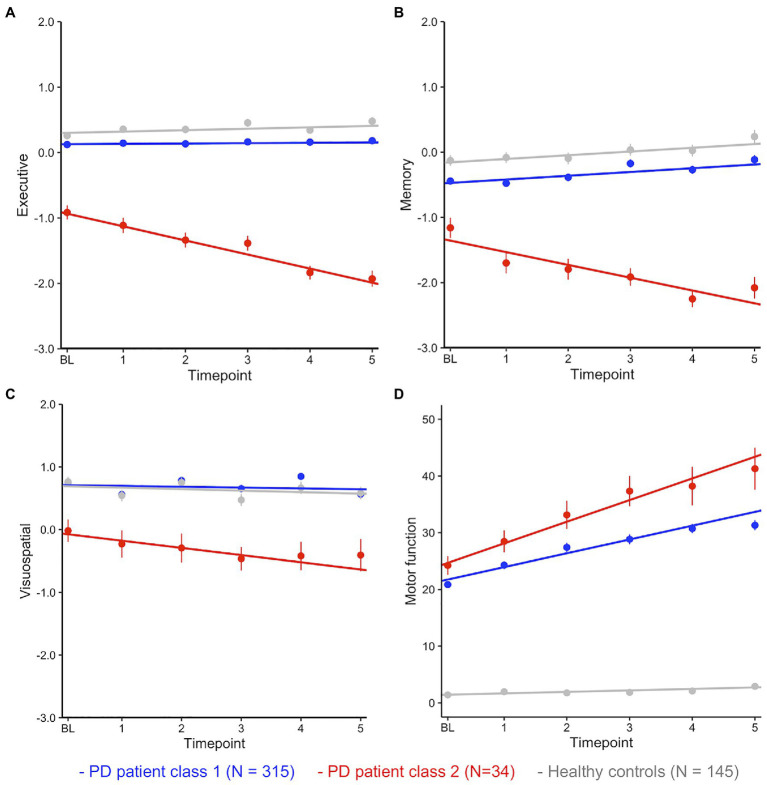
**(A–D)** Mean performance (with SE) at each timepoint by class and cognitive domain. Fitted lines are for a linear model regressing time against cognitive performance for each class.

Plotting of the mean performance scores for each cognitive domain, timepoint, and both classes in Figures 1A–C shows that the smaller patient class (class 2, red) was characterized by performance deficits within 1SD of the larger class (class 1, blue) at baseline but then showed rapid linear decline over time.

Fitting linear mixed effects models with fixed effects of time, class, and time*class in the patients confirmed significant effects of class (i.e., a significant difference between patient classes at baseline) and a highly significant time*class interaction for each cognitive domain (i.e., a significant difference between classes in slopes, Table 3). HC were added (in grey) as a single control group after confirming with a separate multivariate latent class mixed model that cognitive decline across domains was best described as one group for HC (all multi-group solutions had a BIC>4777.33 (1-group), loglik=−2296.67, and 37 parameters).

**Table 3:** Baseline characteristics of PD patients by class and healthy controls.

	PD Class 2 (decliners)	PD Class 1	X2 or *t* (*p*)	HC
N	34	315		145
Age years.	66.06 (7.48)	63.37 (7.46)	1.99 (0.05)	63.96 (7.74)
Male %	79.41	63.81	2.65 (0.10)	65.52
Education years.	15.62 (3.95)	15.53 (2.88)	0.13 (0.90)	15.91 (2.83)
Disease duration (months)	5.65 (5.99)	6.84 (6.70)	−1.09 (0.28)	NA
UPDRS part I score	6.53 (4.33)	5.43 (3.99)	1.42 (0.16)	3.04 (3.06)
UPDRS part II score	6.88 (3.74)	5.65 (4.03)	1.82 (0.08)	0.49 (1.00)
UPDRS part III score	24.24 (9.53)	20.86 (8.68)	1.12 (0.06)	1.41 (2.43)
% Tremor dominant	79.41	70.70	0.76 (0.39)	NA
% PIGD dominant	14.71	19.43	0.19 (0.66)	NA
HoehnandYahr, % st 2	64.71	55.87	0.65 (0.42)	NA
UPSIT, % hyposmia	97.06	91.11	0.75 (0.39)	42.76
REM disorder, % yes	61.77	34.30	8.78 (0.003)	18.62
MOCA, % with <26	44.11	21.27	7.69 (0.006)	NA
GDS-15, % normal	70.59	88.25	6.77 (0.009)	91.72
Any QUIP disorder, %	26.47	19.43	0.55 (0.45)	20
STAI trait total score	46.41 (4.40)	45.99 (4.16)	0.54 (0.60)	46.57 (3.37)
STAI state total score	47.12 (5.59)	47.46 (5.20)	−0.33 (0.74)	47.83 (5.17)
APOE4 presence, %	31.03	24.38	0.32 (0.57)	24.81
CSF α-synuclein	1363.60 (542.85)	1550.18 (681.59)	−1.83 (0.08)	1739.65 (769.08)
amyloidß42	679.50 (307.88)	938.27 (415.66)	−4.41 (<0.001)	1011.77 (507.34)
t-tau	182.80 (72.68)	169.83 (54.70)	0.97 (0.34)	197.01 (83.07)
p-tau	16.75 (7.08)	14.77 (5.06)	1.45 (0.16)	18.12 (8.98)

MDS-UPDRS, Movement Disorders Society Unified Parkinson’s Disease Rating Scale; PIGD, postural instability-gait disturbance; UPSIT, University of Pennsylvania Smell Identification; REM, rapid eye movement; MOCA, montreal cognitive assessment; GDS-15, Geriatric Depression Scale-15; QUIP, Questionnaire for Impulsive-Compulsive Disorders in *Parkinson’s* Disease; STAI, State and Trait Anxiety Inventory; and CSF, cerebrospinal fluid marker. *p* values and the respective statistic (*X*^2^ for categorical variables, *t* for continuous variables) are reported for the comparison class 2 vs. class 1. NA, by definition not applicable for healthy controls.

*Post-hoc* comparisons between the large PD class (class 1) and HC showed that, on average, patients performed worse than HC in EF and memory but did not differ in performance on the visuospatial task (Figure 1; Table 3). Compared to the large performance decrements of patients in class 2, the performance deficit in class 1 patients was small, with an estimated difference of less than half a SD at baseline. Interactions between class and time were not significant (all *ps*>0.05). In summary, these analyses suggest longitudinal trajectories of cognitive change in PD are best described by two distinct latent classes: (1) A large group of patients that performed worse (compared to matched HC) on tests of EF and episodic memory but remained stable over 5years, suggesting subtle cognitive impairment is common in PD. (2) A small group of PD patients with rapid and pronounced loss of global cognitive functions across time.

For comparison, class differences in motor function (UPDRS score part III) were estimated over time (Figure 1D; Table 3). Here, both groups showed large differences to HC at baseline and over time. The rapid decliners also showed pronounced loss of motor functions over time, however they did not differ significantly from class 1 patients at baseline (*p*=0.08, *cf.* section Class Comparison on Baseline Demographics, Clinical Variables, and CSF Biomarkers).

### Class Differences in Medication and Drop-Out

At the first follow-up, 63.03% of patients in the large class (class 1) were on anti-PD medication, compared with 56.67% among the rapid decliners [*χ*^2^ (df=1, *N*=349)=0.23, *p*=0.63]. At the second follow-up, the number of patients on medication was over 87% in both classes and over 90% at subsequent visits, suggesting differences in cognitive decline were not due to differences in medication.

Table 4 shows the number of follow-up visits for each class. The majority of patients in both classes participated in all five follow-up visits and there was no significant difference in the proportion of maximum follow-up visits (i.e., 5) between classes [*χ*^2^ (df=1, *N*=349)=2.82, *p*=0.09], suggesting that study drop-out had no impact on the results.

**Table 4:** Indices of fit multivariate latent class mixed model with 1–4 classes.

G	npm	loglik	BIC	% class 1	% class 2	% class 3	% class 4
1	37	−5618.56	11453.76	100			
2	44	−5585.80	11429.22	90.26	9.74		
3	51	−5572.93	11444.48	88.83	2.29	8.88	
4	58	−5569.60	11478.79	81.94	1.14	7.45	9.46

G, number of estimated groups; Loglik, log likelihood; npm, number of parameters; and BIC, Bayesian Information Criterion.

### Class Comparison on Baseline Demographics, Clinical Variables, and CSF Biomarkers

In order to identify clinical predictors of rapid cognitive decline at baseline, *post-hoc* comparisons between the patient classes revealed that the rapid decliners were more likely than the rest of the patients to score below 26 on the MOCA, be mildly depressed, suffer from a REM sleep disorder, and have lower levels of CSF amyloidß42 level (Table 1) though these differences should be interpreted with caution as only the differences in amyloidß42 level reached significance after control for comparisons of 22 different variables.

In terms of the potential of these markers as stand-alone clinical screening tools at baseline, it should be noted that the majority of patients in both classes had a normal MOCA score of 26 or higher at baseline, suggesting that brief clinical screeners at baseline do not accurately identify rapid decliners. Prevalence of depression did not differ between that large patient class 1 and HC [*χ*^2^ (df=1, *N*=349)=0.92, *p*=0.34], which indicates that a higher prevalence of depression may be specific to the rapid decliners and not PD in general. Nevertheless, only 10 out of 34 patients were considered mildly depressed, and no patient among the rapid decliners was judged to be moderately or severely depressed.

Amyloidß42 level was significantly lower in the rapid decliner at baseline whereas amyloidß42 level did not differ between the large patient group and HC [*t*(225.50)=−1.50, *p*=0.14].

Interestingly, neither demographics, such as age, gender, and education, nor motor symptoms, disease severity or disease duration were strongly predictive of rapid cognitive decline {all *p*s<0.05, though we note trend level differences in age [*t*(40.42)=−1.98, *p*=0.054] and UPDRS part III motor score [*t*(39.17)=1.99, *p*=0.054] between the patient classes}. Because of the large number of independent comparisons, trends of *p*>0.06 are not considered further.

### Dopamine Transporter Loss and Rapid Cognitive Decline

**Figure 2:**
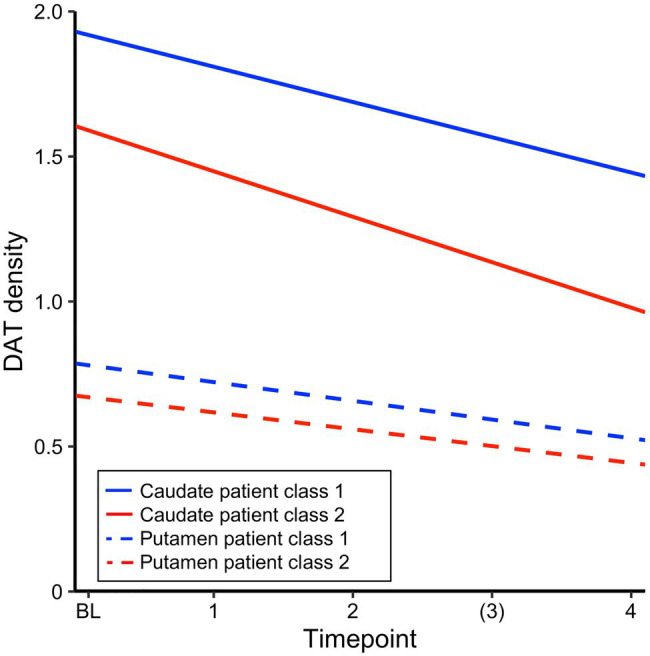
Estimated intercept and slopes of dopamine transporter (DAT) density for each patient class from a linear mixed effects model with time and class, run separately for caudate and putamen.

Differences in regional neuropathology between the large PD class and the rapid decliners, estimated as DAT density in caudate and putamen, were compared between groups. DAT density estimates from SPECT scans obtained at the screening visit were available for 305 patients from the larger class and 33 patients from the class of rapid decliners and 293 and 28 patients, respectively, had at least one follow-up scan. Linear mixed effects models with DAT density as the outcome and class and time as predictors were fitted separately for caudate and putamen. For caudate DAT density, the results revealed a significant effect of time, class, and a trend for a significant time*class interaction, suggesting slightly accelerated loss of caudate DAT density for the rapid decliners. For putamen DAT density, a significant effect of time, class but no significant time*class interaction was found (Table 5). Figure 2 illustrates the main effects and interactions for both models. A linear model assessing the interaction between region (caudate/putamen) and class for baseline DAT level confirmed that a difference in DAT density between classes at baseline was pronounced for caudate relative to putamen (interaction Estimate=−0.22, *SE*=0.11, and *p*=0.05). Together, this suggests a highly significant DAT deficit for the rapid decliners that was marginally more pronounced for caudate both at baseline and in terms of loss over time. For comparison, the reduction of CSF biomarker levels over time did not differ between classes for myloidaß42 (Table 5), t-tau, or p-tau (*ps*>0.50). Of note, there was a significant interaction between class and time for α-synuclein (Estimate for time*class=67.36, *SE*=33.34, and *p*=0.04), but in the direction that α-synuclein increased at follow-up timepoints to the level of the comparison class (i.e., regression to the mean), suggesting that the marginal difference at baseline may be a false positive. Thus, unlike DAT, CSF biomarker change did not resemble the pattern of cognitive decline.

**Table 5:** Linear mixed effects models for DAT and amyloidß42.

Marker	Effect	Estimate	*SE*	*p*
*Comparison of PD class 1* vs. *PD group 2*				
Caudate DAT	Time	−0.16	0.02	<0.001
	Class	−0.33	0.09	<0.001
	Class*Time	−0.04	0.02	0.06
Putamen DAT	Time	−0.06	0.01	<0.001
	Class	−0.11	0.05	<0.001
	Class*Time	0.01	0.01	0.51
amyloidß42	Time	−18.81	17.31	<0.001
	Class	−254.80	72.28	<0.001
	Class*Time	−7.14	18.06	0.69

Within the group of rapid decliners, change in caudate DAT was associated with change in visuospatial ability (estimated with individually fitted slopes for each patient and each outcome, *r*=0.42, *p*=0.03, and *N*=28), but not with change in executive functions or memory (*p*>0.05).

## Discussion

Cognitive deficits in PD are common but incompletely understood. In the current study, we used a data-driven clustering approach to identify latent groups of cognitive decliners in PD. With this method, the large majority of the patients (90%) was statistically best described as a single group who showed slight cognitive impairment compared to matched controls but remained stable over the first 5years of the disease. A small group (10%) of rapid decliners showed a marked loss in all cognitive domains over 5years, up to 0.25SD each year in comparison to the rest of the patients. Of particular note, the rapid decliners performed lower but within 1SD at baseline. Similarly, the majority of rapid decliners had normal (> 26) MOCA scores at baseline. Moreover, they did not differ significantly from the large sample in age, sex, education, mean disease duration, or motor symptom severity at baseline (though we note a trend for age and UPDRS motor score at baseline and accelerated decline in motor function over time).

Prior work that has taken into account non-motor symptoms such as cognitive deficits has revealed quite a different pattern of subtypes in PD, even using the same PPMI sample. Fereshtehnejad et al. (2015, 2017) for example, showed that at baseline, younger patients with predominant motor symptoms form a mild disease sub-type on one end of the clinical spectrum whereas a small group of “diffuse-malignant” symptoms including cognitive deficit represent a more aggressive clinical sub-type. Focusing in on the baseline cognitive assessment in PPMI as the basis for grouping, LaBelle et al. (2017) identified up to six sub-types. Again, and unlike our rapid decliners, global deficits in cognition at baseline were strongly associated with differences in demographics and symptom severity in other domains. While such baseline groupings may, to some degree, be predictive of future progression of disease on the mean group level, individual differences in trajectories are by design not considered in these classifications. In comparison, our approach identified a homogeneous group of rapid decliners but did not take baseline predictors into account *a priori*. Thus, our results and those of baseline groupings are not in conflict with each other but should be viewed as providing complementary information. Our results highlight a rare but aggressive form of cognitive decline, as well as accelerated loss of motor function, in PD that may be missed using only baseline assessments. However, this is not to say that the larger comparison group of patients in our analysis is not composed of meaningful sub-groups of patients in terms of baseline disease severity.

The measures that were most predictive of rapid cognitive decline in PD were higher prevalence of mild depression, REM disorder, lower levels of CSF amyloidß42, and lower DAT density on SPECT, particularly so in caudate. Moreover, caudate loss across time was proportional to loss of cognitive deficits in the decliners, though only on the visuospatial test. According to anatomical tracing and human fMRI functional connectivity, the caudate is predominantly connected to prefrontal and other associative cortical areas whereas the putamen is in large parts connected with motor areas (Provost et al., 2015). Consistent with this, prior imaging work in PD patients has identified that DAT loss in putamen is proportional to decreases in midbrain-putamen fMRI connectivity (Rieckmann et al., 2015) and that altered fMRI activation in putamen correlates with motor impairment (Herz et al., 2014). Conversely, cognitive impairment in PD has been associated with disrupted connectivity in brain networks subserving higher-order associative functions (Lopes et al., 2017). In line, depression in PD has been linked to lower extrastriatal neurotransmitter functions in PD (Remy et al., 2005), though higher prevalence of depression may also indicate subjective awareness of impending cognitive decline (Burmester et al., 2016). Lower baseline amyloidß42 levels in the rapid decliners also point toward a high load of cortical “Alzheimer like” pathology (e.g., Hansson et al., 2018), collectively suggesting that early neuropathological changes are not confined to motor areas in these patients but extend to association cortex.

Interestingly, prior work has reported reduced caudate DAT and increased amyloid burden as variables that distinguish PD from dementia with Lewy bodies (DLB; e.g., Gomperts et al., 2016). A high prevalence of REM sleep disorder is now also established as a hallmark symptom of DLB (McKeith et al., 2017). However, a key diagnostic criterion for DLB is the diagnosis of dementia before or concurrent with Parkinsonism. As discussed above, this was not the case for the rapid decliners who only showed mild cognitive deficits at baseline. Our work clearly highlights an urgent need to continue research on the diagnostic criteria that accurately distinguishes subtypes of synucleinopathies and develop multivariate prediction models at time of diagnosis that may forecast the likely course of disease.

A recent study from PPMI identified that caudate DAT loss was present in as many as 50% of patients at baseline (Pasquini et al., 2019). Even though our study confirmed these findings in terms of caudate DAT loss being a predictor of worse cognition, our group of rapid decliners was much smaller, suggesting heterogeneity in cognitive trajectories among individuals with pronounced DAT loss. On the one hand, the differences between the prevalence of caudate DAT loss at baseline and rapid cognitive decline are interesting, and future studies should focus on additional risk or protective factors that link caudate loss to cognitive decline. On the other hand, this discrepancy may suggest that our statistical approach misses more subtle impairment within individuals at risk. Pertaining to this point, data from large samples of healthy individuals have suggested that normal aging is also characterized by sub-groups with differing cognitive trajectories (e.g., decliners vs. maintainers; Josefsson et al., 2012), yet our model was unable to detect different cognitive subtypes of change in our healthy control sample. Thus, there is a possibility that the model is not sensitive enough to recognize a finer-grained picture of subtypes in PD cognition even though these may be clinically meaningful.

Another limitation of the current study for accurately describing cognitive change in PD is the short follow-up time in the PPMI. Cognitive change often occurs gradually over many years of the disease and is particularly prevalent in the late stages. Thus, our current analyses cannot identify whether these groups would follow the same pattern of stability and decline also over longer periods. In fact, it is likely that the large group of stable patients in our study will become more heterogeneous with time and that important sub-types of disease already exist but are not detected within this relatively short time span of the early stage of the disease. In line with this important caveat, Pigott et al. (2015) showed that, of patients who were enrolled 5years into the disease (i.e., where observations of the current cohort end), the cumulative incidence of cognitive impairment was 9% but increased to almost 50% 6years later. Thus, while the current study contributes to an understanding of early cognitive decline and its predictors, we likely “miss” the period of the disease where cognitive impairment becomes more pronounced and common in the current sample. Related to this point, while drop-out was low for both classes in the current study, non-random drop-out for rapid decliners would likely increase with longer follow-up periods or measurement in later stages of the disease. Thus, follow-up studies of these individuals or studies in independent samples using a similar approach should be designed with appropriate control samples or appropriate statistical control for non-random drop-out. In addition, the current results need to be validated in an independent cohort.

In clinical practice, pronounced PIGD-motor symptoms at the time of PD diagnosis are flagged as a warning sign for a rapid deterioration in motor symptoms and non-symptoms. The current results suggest that there is a subtype of PD patients that will decline rapidly in cognitive functions without presenting with severe deficits in motor- or other symptoms at the time of diagnosis. Our results further indicate that approximately every 10th individual among the newly diagnosed PD patients without dementia at the time of diagnosis belongs to this subtype of rapid cognitive decliners, making them relatively rare and maybe easily overlooked in a clinical setting. The current study thus highlights the need for clinicians to prioritize an elaborated and repeated neuropsychological assessment as a general routine.

## Data Availability Statement

Publicly available datasets were analyzed in this study. This data can be found here: https://www.ppmi-info.org/access-data-specimens/download-data/.

## Ethics Statement

The studies involving human participants were reviewed and approved by the appropriate local ethics committees of each site of the PPMI. The patients/participants provided their written informed consent to participate in this study.

## Author Contributions

AR and SA conceived the study. SA, MJ, LS, and AR contributed significantly to the analysis of the data and drafted or revised a significant portion of the manuscript. All authors contributed to the article and approved the submitted version.

## Funding

The work was supported by the Strategic Research Area Neuroscience Umeå (StratNeuro). SA, LS, and AR were also supported by an ERC Starting Grant from the European Research Council (ERC) under the European Union’s Horizon 2020 research and innovation program (Grant agreement No. 716065) to AR. PPMI – a public-private partnership – is funded by the Michael J. Fox Foundation for Parkinson’s Research and funding partners (https://www.ppmi-info.org/about-ppmi/who-we-are/study-sponsors/).

## Conflict of Interest

The authors declare that the research was conducted in the absence of any commercial or financial relationships that could be construed as a potential conflict of interest.

## Publisher’s Note

All claims expressed in this article are solely those of the authors and do not necessarily represent those of their affiliated organizations, or those of the publisher, the editors and the reviewers. Any product that may be evaluated in this article, or claim that may be made by its manufacturer, is not guaranteed or endorsed by the publisher.
